# Bigels as Delivery Systems of Bioactive Fatty Acids Present in Functional Edible Oils: Coconut, Avocado, and Pomegranate

**DOI:** 10.3390/gels9040349

**Published:** 2023-04-21

**Authors:** Manuela Machado, Sérgio Cruz Sousa, Luís Miguel Rodríguez-Alcalá, Manuela Pintado, Ana Maria Gomes

**Affiliations:** CBQF Centro de Biotecnologia e Química Fina-Laboratório Associado, Escola Superior de Biotecnologia, Universidade Católica Portuguesa, Rua Diogo Botelho 1327, 4169-005 Porto, Portugal

**Keywords:** release properties, carboxymethylcellulose, bioactive compounds, rheology, monoglycerides

## Abstract

Bioactive fatty acids possess several benefits for human health; however, these molecules show a reduced oxidative stability and consequently reduced bioavailability. This work aimed to develop novel bigels as a strategy to protect bioactive fatty acids present in three different vegetable oils with nutritional attributes (coconut oil, avocado oil, and pomegranate oil) during passage through the gastrointestinal tract (GIT). Bigels were prepared using monoglycerides-vegetable oil oleogel and carboxymethyl cellulose hydrogel. These bigels were analyzed in terms of structure and rheological characteristics. According to the rheological properties, bigels exhibited a solid-like behavior since G’ was higher than G”. The results showed that the proportion of oleogel was essential to the viscosity of the final formulation as an increase in this fraction was responsible for an increase in viscosity. The fatty acids profile was evaluated before and after simulated GIT. The bigels protected the fatty acids against degradation; in the case of coconut oil, the reduction of key fatty acids was 3 times lower; for avocado oil, 2 times lower; and for pomegranate oil, 1.7 times lower. These results suggest that bigels can be used as part of an important strategy for bioactive fatty acid delivery for food applications.

## 1. Introduction

Bioactive lipids (such as essential fatty acids, sterols, tocopherols, carotenoids, and fat-soluble vitamins) provide many health benefits when consumed in appropriate amounts. They may act as antioxidants, as immunomodulators, and can improve bone health, eye and brain function, and reduce coronary diseases [1]. Unfortunately, most of these compounds are not synthesized in the human body, and they must be obtained through diet. However, in regular diets, the levels of these bioactive lipids are low. Consequently, many people do not ingest sufficient levels. Moreover, their tendency to oxidize during processing and storage in conjunction with their poor stability and low absorption characteristics within the human gut are also responsible for their poor levels in the general population’s diet [2,3].

Considering these facts, the food industry is seeking new methods to fortify foods with more stable and bioavailable forms of these bioactive lipids, particularly fatty acids. In this context, bigels appear to present interesting strategy, because they combine the characteristics of organogels and hydrogels, which make them capable of delivering both hydrophilic and lipophilic bioactive compounds [4]. Technically, bigels are semi-solid formulations obtained mainly through high-speed mixing of organogels and hydrogels at specific temperatures [5,6,7]. In addition to their unique thermodynamic behavior, they also allow for custom tuning of bigels based on the viscoelasticity and the variety of materials that can be used to formulate them. The use of these gels can provide relevant opportunities for the development of nutritionally enriched foods. These systems have been previously studied for their ability to release controlled active compounds such as omega-3 fatty acids, CoQ_10_, β-carotene, and lycopene and thus show potential for the delivery of bioactive fatty acids [8,9,10,11].

The incorporation of bioactive fatty acid sources in functional foods faces several challenges due to the high susceptibility of fatty acids to oxidation, which causes rancidity (off-flavors), consumer rejection, and a decrease in nutritional value [12]. In this context, the present work aims to develop and characterize bigel properties containing three different vegetable oils (coconut, avocado, and pomegranate) and evaluate their capacity to deliver their main bioactive fatty acids. These vegetable oils possess distinct lipid compositions. Coconut oil is rich in medium-chain fatty acids, which play a relevant role in obesity management due to their impact on thermogenesis; avocado oil is rich in oleic acids, which show interesting anti-inflammatory potential; and pomegranate oil is the major source of conjugated linolenic acids, with high potential in obesity prevention, due to their capacity to modulate adiponectin and leptin secretion. The use of three different lipid compositions enables more consolidated knowledge and validated robustness of the bigel’s potential as a protective strategy for lipid delivery.

## 2. Results and Discussion

### 2.1. Optimization of Bigel Production

**Figure 1:**
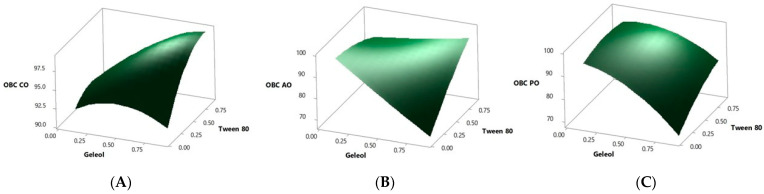
Response surfaces for oil-binding capacity (OBC) optimization ((**A**)-coconut oil, (**B**)-avocado oil, and (**C**)-pomegranate oil).

To investigate the impact of gelling agents Tween 80 and geleol on oil binding capacity, an experimental design was applied. The results obtained (Table 1) showed that the produced bigels had an oil binding capacity ranging between 92.39% and 98.66% for coconut oil, 72.64% and 96.67% for avocado oil, and 74.76% and 97.74% for pomegranate oil. The fit model *p* values are related to the importance of gelling agents in oil binding capacity. For coconut oil, Tween 80 had a higher *p*-value (0.001) than geleol (0.000), which means that a higher percentage of oil binding capacity can be obtained using a higher quantity of Tween 80. In the case of avocado oil, both gelling agents contributed equally to the oil binding capacity as both presented the same *p*-value. Regarding the pomegranate oil, high percentages of oil binding capacity can be achieved when increasing the Tween 80 quantity (Figure 1C). The mathematical models obtained for each response surface were used to perform a multi-objective optimization. The goal of the optimization was to determine the best ratio of Tween 80 and geleol to maximize the oil binding capacity of each edible oil tested. Regarding coconut oil, the model predicted a 99.16% oil binding capacity with a 95% confidence interval (that can vary between 96.94 and 101.39) using 0.87 g of Tween 80 and 0.90 g of geleol. In the case of avocado oil, 97.30% of oil binding capacity can be achieved with 95% confidence (varying between 91.75 and 102.85) using 0.10 g of each gelling agent (1:1). Finally, for pomegranate oil, the maximum oil binding capacity (96.70%) can be obtained using 0.56 g of Tween 80 and 0.13 g of geleol, and the values of oil binding capacity can vary between 93.28 and 100.13 with 95% of the confidence interval. Interestingly, different quantities of Tween 80 and geleol were required to reach maximum oil binding capacity (the highest amounts of each were required in the case of coconut oil—almost 1 g of each—and the lowest amounts of each were required in the case of avocado oil) as well as different proportion ratios. While small ratios were reported for coconut oil and avocado oil (almost 1:1 and 1:1, respectively), in the case of pomegranate oil, the proportion of Tween 80 was four-fold higher than geleol. Small ratios were associated with a faster release whereas large ratios sometimes led to a more controlled release of delivered compounds [13]. Validation of the predictive models was performed in triplicate for each oil. The experimental oil binding capacity values were within the limits established in the confidence intervals. Thus, the optimized bigels showed 99.06 ± 0.56% of oil binding capacity for coconut oil, 93.61 ± 0.33 for avocado oil, and 95.57 ± 1.38% for pomegranate oil. In the literature, there are no data using this bigel structure for delivery of this type of fatty acid. The available data include the use of coconut oil or pomegranate oils as part of the organogel fraction in order to enhance the delivery of lipophilic molecules such as curcuminoids or as a structure agent for fat replacers [14,15,16].

### 2.2. Structural Characterization of Bigels

**Figure 2:**
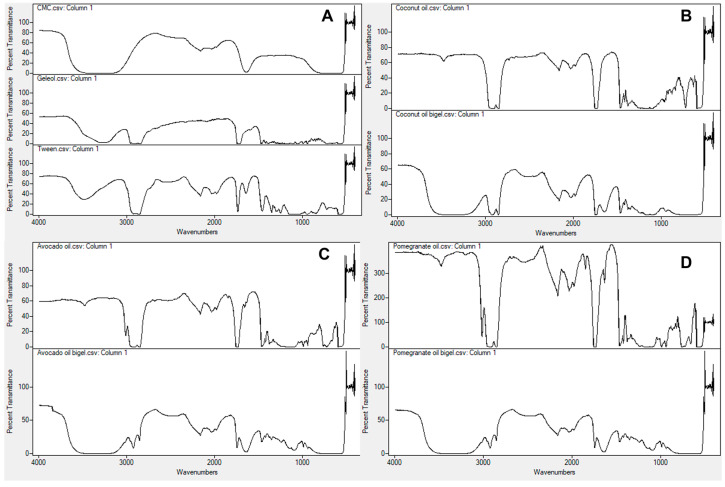
FTIR spectra of bigel formulation components: (**A**), coconut oil and corresponding bigel (**B**), avocado oil and their bigel, and (**C**), pomegranate oil and corresponding bigel (**D**).

The FTIR spectra of bigels were analyzed to assess the interactions between the oleogel and the hydrogel within the bigel system. As shown in Figure 2, a broad band at 3750–3000 cm^−1^ was observed for all bigels and could be associated with the presence of O–H stretching vibrations participating in hydrogen bonds within CMC and the heads of the acyl groups present in the oleogel [10,17]. The absorption peaks between 2928 and 2932 cm^−1^ represented the stretching vibration of C-H and HC=CH bonds and correspond to the presence of unsaturated and saturated fatty acyl chains characteristic of each of the vegetable oils under study [10]. The higher intensity of these peaks in coconut oil bigel spectrum can be due to the high concentration of geleol used in its formulation. In this formulation, another peak characteristic of the stretching vibration of C-H bonds in saturated fatty acyl chains was observed at 1468 cm^−1^ or 1470 cm^−1^ in coconut bigel and geleol spectra, respectively. The absorption peaks observed at 1746 cm^−1^ in the coconut oil bigel spectrum, and at 1750 or 1752 cm^−1^ in the pomegranate and avocado oil spectra, respectively, indicated the presence of the C=O stretching vibration of the ester group in vegetable oil glycerides [14,18,19]. The differences found between the coconut oil bigel and the pomegranate and avocado oil bigels can be related to the high amount of geleol used in the former for its preparation, as mentioned above. In addition, this may also be related to the high amount of oil retained in the bigel. Such influence of the concentration and proportion of bigel components on their FTIR spectra have been shown for other bigel systems, for example, for fish-oil-based [20] and sunflower-based [21] bigel systems. The band between 1660 and 1650 cm^−1^ was present in all bigel spectra regarding the stretching vibration of the carboxyl group (COO-) of CMC [17]. The bands between 1300 and 1000 cm^−1^ observed in the coconut and pomegranate bigel spectra can be associated with the C-O stretching vibration in the ester group [14,18,19]. According to these results, there appear to be no interactions between the oils and polymers as the main absorption peaks remained.

### 2.3. Rheological Properties

**Figure 3:**
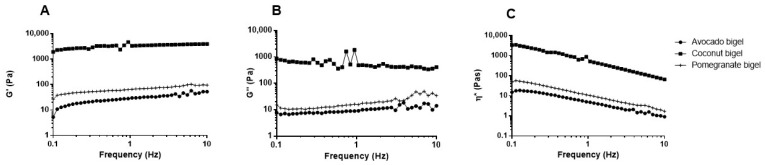
Rheological properties of bigels: (**A**) elastic modulus (G’), (**B**) viscous modulus (G”), (**C**) complex viscosity (ƞ*), avocado oil (●), coconut oil (▪), and pomegranate oil (⁺).

The results (Figure 3) showed that the produced bigels presented differing rheological profiles, with coconut oil bigels presenting higher elastic modulus (G’), viscous modulus (G”), and complex viscosity (ƞ*) compared to the bigels produced with avocado and pomegranate oils. This can be related to the amount of geleol used in the coconut oil bigel preparation (9 times higher than the amount used in the other formulations). When comparing the avocado oil and the pomegranate oil bigels, the results showed that the former had slightly higher values for all parameters than the latter. This result can be explained by the higher Tween 80 content present in the pomegranate oil bigel. According to these results, a positive relationship between the increase in gelators and the increase in viscosity is apparent. Moreover, an increase in the oleogel fraction led to an increase in viscosity, with the same trend being reported by other authors [4,9]. In all bigels, the viscosity decreased with the frequency increase. The prevalence of G’ relative to G” is an indicator of the gel strength. G’ and G” do not intercept (no point of G’ is equal to G”) at the studied frequencies, which may suggest that the bigel does not show gel-to-sol transformation. This fact can indicate the presence of stronger internal forces in the bigel, which represent a solid-like behavior for this matrix [6,22].

**Figure 4:**
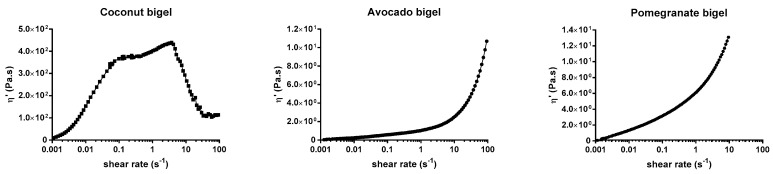
Viscosity curves of bigels vs. shear rate.

For the viscometry measurements results (Figure 4), coconut oil bigel showed a different behavior in comparison to the others as it presented up to 4.7 s−^1^ increases in the instantaneous viscosity (ƞ’), after which point a decrease was observed. With the increase in the shear rate, the ƞ’ decreased. In contrast, the increase in shear rate increased the ƞ’ of avocado and pomegranate oil bigels. This difference in behavior of coconut oil bigel can be attributed to the high oleogel fraction. A previous work using polymer fish oil bigels demonstrated that an increase in the oleogel fraction resulted in a decrease in the apparent viscosity [8].

### 2.4. In Vitro GIT Survival of Bioactive Fatty Acids within Bigels

In vitro digestion was used to study the potential of produced bigels to reduce the degradation of bioactive fatty acids during passage through the gastrointestinal tract (GIT). The fatty acids profile of the bigels and respective oils before and after GIT is presented in Table 2.

**Figure 5:**
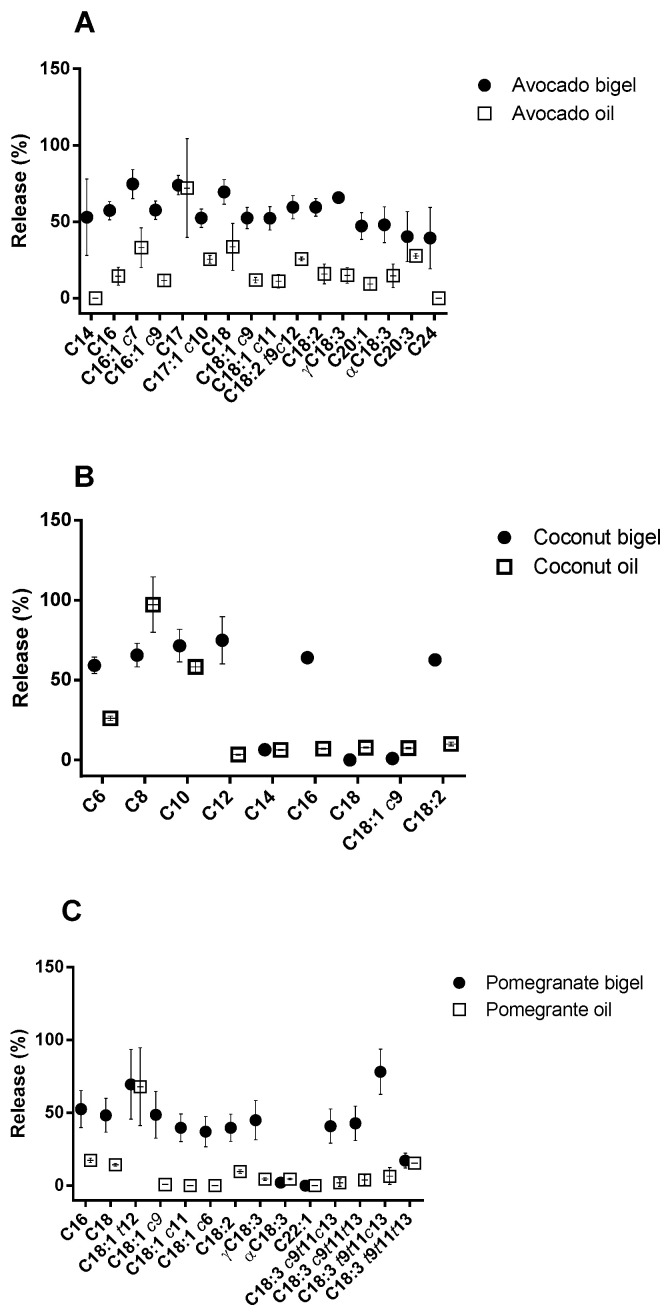
GIT release percentages of the main bioactive fatty acids found in the different vegetable oils: (**A**) coconut oil, (**B**) avocado oil, and (**C**) pomegranate oil.

Significant differences (*p* < 0.05) were obtained for all tested samples. In the case of coconut oil, the fatty acid content was reduced by 87.9% after GIT (this percentage was much lower when coconut oil was delivered as a bigel (27.7%)). Regarding avocado oil, there was a degradation of 86.7% of fatty acid content during GIT. As verified for coconut oil, the use of bigel led to a lower degradation percentage of 44.2%. The polyunsaturated nature of pomegranate oil makes it more susceptible to oxidation, as can be seen from the 96.3% reduction in total fatty acid content. The formulated pomegranate oil bigel reduced this percentage to 56.6%. According to the fatty acids profile results, the release percentages of the main bioactive fatty acids were calculated. As shown in Figure 5B, for coconut oil, the main bioactive fatty acids release percentages were higher in bigel formulation. Around 75% of lauric acid C12 (the main bioactive fatty acid in coconut oil) reached the intestine, a value which was 25 times higher than the value obtained for oil after digestion (3.48 ± 0.33%). The health benefits of coconut oil are related to its high quantities of medium-chain fatty acids (C8, C10, and C12) [23], and it was proven that the incorporation in the bigel enabled the protection of the fatty acids since there was an increase in the fraction that reached the intestine.

Regarding the formulation with avocado oil, a positive effect of the bigel was also observed (Figure 5A). Oleic acid (C18:1 *c*9) is the main fatty acid present in this oil, and it is responsible for its main putative potential health benefits [24]. In the formulation of bigels, their release percentage reached 50%. This percentage was 4 times higher than the value obtained for pure avocado oil after digestion (12%). Other important fatty acids in avocado oil are linoleic (C18:2 *c*9*c*12) and linolenic acids (C18:3) whose release percentages in bigels were 2 and 3 times higher, respectively, when compared to avocado oil.

Pomegranate oil is an important source of polyunsaturated fatty acids, particularly conjugated linolenic fatty acids (CLNAs), which have several beneficial effects on human health; however, this compound has reduced stability [25,26]. The incorporation of pomegranate oil into bigels led to a reduction in CLNA degradation during the GIT passage when compared to the pure oil after digestion (Figure 5C). About 40% of punicic acid-C18:3 *c*9*t*11*c*13 (the main bioactive CLNA present in this oil) reached the intestine. In contrast, only 2% of this pure oil fatty acid reached the intestine after digestion. In addition, higher release percentages were also observed for other important CLNAs (α-eleostearic acid C18:3 *c*9*t*11*t*13, catalpic acid C18:3 *t*9*t*11*c*13, and β-eleostearic acid C18:3 *t*9*t*11*t*13).

**Figure 6:**
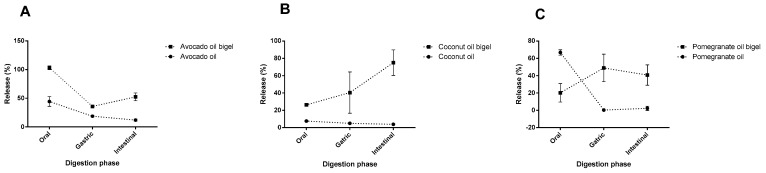
Evolution of main bioactive fatty acid release (%) during gastrointestinal digestion. (**A**) avocado oil and correspondent bigel, (**B**) coconut oil and coconut oil bigel, and (**C**) pomegranate oil and correspondent bigel.

The evolution of the release of the main bioactive fatty acid from each oil during digestion (Figure 6) verified that the release increased after the gastric phase in the bigel formulations. On the other hand, in free-form oils, the release remained similar between the gastric and intestinal phases. In all digestion phases, the amount of the main bioactive fatty acids of each oil remained higher in bigel formulations compared to the correspondent oil.

According to these results, bigels can be a valuable strategy to protect different bioactive fatty acids during passage through the GIT. Although there are no similar studies using bigels as protective delivery systems for such oils, several studies point to the positive effect of bigels as delivery systems for lipophilic compounds such as lycopene, β-carotene, vitamin E, and Coenzyme Q10, given their composition and unique structure [9,10,11,13]. For example, in the case of lycopene, a bigel produced with high acyl gellan gum and glycerol monostearate (GMS)-beeswax based oleogel enhanced the release of lycopene in the intestine. Moreover, the high ratios of oleogel can retard the lycopene release, which can be an advantage for delivery of fat-soluble nutraceuticals, extending their residence time in the body [10]. Regarding β-carotene, it was incorporated in a bigel based on κ-carrageenan hydrogel and monoglyceride oleogels to improve their stability during GIT passage. The authors concluded that the bigel modulated the release of β-carotene since minor release percentages were observed in simulated gastric fluid, and the release ratios increased in the simulated intestinal fluid [9].

## 3. Conclusions

Bigels can be a valuable delivery system for different classes of bioactive fatty acids with high nutritional value. In the current study, a bigel delivery system was used for the first time for delivery of bioactive fatty acids from coconut, avocado, and pomegranate oils. The oils structured in bigels showed high GIT release percentages when compared to pure oils, which can enhance their biological potential and allow for targeted delivery in the intestine. Moreover, the high release percentages were also traduced in the enhanced amount of essential fatty acids after GIT. At this point, it is important to understand the bioactivity of the loaded bigels in the intestine in order to provide a blueprint for the future development of bioactive lipids delivery and protection structures. The rheological characteristics of produced bigels can be easily modulated by alterations in the organogel/hydrogel ratio, presenting different opportunities to protect and deliver lipids in food industry applications.

## 4. Materials and Methods

### 4.1. Materials and Reagents

Geleol was kindly donated by Gatefossé (Saint-Priest, France). Avocado oil was provided by Fula (Algés, Portugal), pomegranate oil was obtained from All Organic Treasure (Wiggensbach, Germany), and coconut oil, from Origens Bio (Arruda dos Vinhos, Portugal). Carboxymethylcellulose was obtained from Merck (St. Louis, MO, USA). Tritridecanoin and punicic, catalpic, α and β- eleostearic acids standards were obtained from Larodan (Solna, Sweden); HPLC grade methanol, hexane, and dimethylformamide (DMF) were obtained from VWR (Radnor, PA, USA); and sodium methoxide was procured from Acros Organics (Morris Plains, NJ, USA). The standards Supelco 37 and CRM-164, Tween 80, and sulfuric acid were obtained from Sigma (St. Louis, MO, USA).

### 4.2. Experimental Design, Modelling, and Optimization

In this work, a response surface methodology was used to evaluate the effect of gelling agents on the oil binding capacity of bigels containing bioactive oils. The selected factors were the amount of geleol and Tween 80. The experimental design was based on a central composite design with two factors with two levels (coded as −1 and 1) composed of 8 cube points and 6 center points in the cube. The design was performed using Minitab software. Table 1 shows factors and levels of each studied variable and the analyzed response (oil binding capacity). All the experiments were performed randomly. Data were fitted with a quadratic regression. An ANOVA for the response surface quadratic model was used to determine the statistical significance of the model factors. To determine the best oil binding capacity (OBC), a multi-objective optimization procedure based on the desirability function was applied using the same statistics software.

### 4.3. Bigel Preparation

Geleol was melted at 60 °C using a hotplate before being mixed with pre-heated (to 60 °C) Tween 80 (0.9 g/0.86 g for coconut oil, 0.1 g/0.1 g for avocado oil, and 0.12 g/0.56 g for pomegranate oil). Carboxymethylcellulose (2% *w/v*) was added under continuous mixing (2 min). After this step, 5 g of vegetable oil were added, and the mixture was homogenized (1 min at 18,000 rpm) in an ultra-turrax (IKA T 25 digital, Janke and Kunkel IKA-Labortechnik, Germany) and then sonicated (Sonics Vibra-Cell™ VCX 130) at 60% amplitude for 1 min. Following this, the bigels were cooled and stored at room temperature until use.

### 4.4. Oil Binding Capacity

Oil binding capacity was determined as follows. A total of 1 g of bigel was placed in pre-weighed centrifuge microtubes and weighed (*m*1). The tubes were then centrifuged at 4 °C and 15,000 rpm for 20 min, and the excess oil was decanted. The total mass of the tube with the remaining bigel (*m*2) was weighed. The oil binding capacity was calculated according to the following equation:

(1) $$\mathrm{OBC}\;\%=\left[1-\frac{\left(m1-m2\right)}{m1}\;\right]\times 100.$$

Surface optimization was used to select the best ratio of geleol/Tween 80 for an enhanced OBC. The optimized formulations were used in the following analysis.

### 4.5. ATR-FTIR Spectra

Fourier transform infrared (FTIR) was performed using a Spectrum 100 FTIR Spectrometer (Perkin Elmer, Waltham, MA, USA) equipped with the attenuated total reflectance (ATR) mode. The spectra were recorded from 4000 to 500 cm^−1^ at a resolution of 4 cm^−1^, and an average of 32 scans was reported. These spectra were subtracted from the background spectrum.

### 4.6. Rheological Measurements

Analyses were performed to determine the rheological behavior of the samples. Measurements were conducted on a Gemini Advanced Rheometer (Bohlin Instruments, Cirencester, UK) coupled with a Peltier unit for temperature control, using a stainless-steel cone-and-plate geometry probe CP (40 mm diameter, 4° angle). Samples were placed on a sampling plate, and analysis was performed, using a 1000 µm gap for coconut oil bigel and 150 µm for avocado and pomegranate oil bigels. Elastic and viscous moduli (G’ and G”, respectively) and complex viscosity (η*) were determined as a function of frequency, which varied between 0.1 and 100 Hz, using a strain of 0.1%, which was previously determined to be within the linear viscoelastic region (LVER). Viscometry measurements (shear rate = 0.001 to 100 s^−1^) using a 150 µm gap were conducted to study the shear behavior of the bigel samples. All samples were analyzed in triplicate.

### 4.7. In Vitro Gastrointestinal Tract Simulation

In vitro digestion was performed according to the INFOGEST method [27], using 5 g of bigels or pure edible oils. To screen the impact of gastrointestinal tract (GIT) on fatty acids release, 0.5 mL aliquots of the sample were collected in all GIT phases, and the fatty acids profile was determined. The in vitro digestion was performed in triplicate for each sample.

### 4.8. Bigels Fatty Acids Profile

The bigels’ (150 mg of sample or 500 µL of digested sample) and correspondent oils’ (15 mg) fatty acid profile was evaluated via gas chromatography (GC) after transesterification according to the method described by Machado et al. 2022 [28]. GC analyses were performed in a gas chromatograph Agilent 8860 (Agilent, Santa Clara, CA, USA) with a flame ionization detector (FID), using a BPX70 capillary column (60 m × 0.25 mm × 0.25 μm; SGE Europe Ltd., Paris, France). The following operating conditions were employed. Injector (split 25:1) and FID temperatures were 250 °C and 275 °C, respectively. Hydrogen (carrier gas) was used at a flow rate of 1 mL/min (20.5 psi). The oven temperature program started at 60 °C (held for 5 min), raised 15 °C/min to 165 °C (held for 1 min), and finally 2 °C/min to 225 °C (held for 2 min). Supelco 37 and individual standards from CLNA were used for the identification of fatty acids. The GC analysis was performed in triplicate.

### 4.9. Statistical Analysis

All results are presented as mean ± standard deviation. The data normality was assessed using the Shapiro–Wilk test. Statistical significances between samples were tested at a 0.05 significance level using a one-way analysis of variance (ANOVA) followed by a multiple comparisons test (Tukey) using Minitab software (version 17, LCC, USA).

## Figures and Tables

**Table 1:** Experimental design for Geleol and Tween 80 levels and the results obtained for oil binding capacity concerning the three oils (CO-coconut oil, AO-avocado oil, and PO-pomegranate oil).

Run Order	Pt Type	Blocks	Geleol	Tween 80	OBC CO	OBC AO	OBC PO
1	0	2	0.50	0.50	97.24	88.37	91.79
2	0	2	0.50	0.50	97.35	88.35	91.71
3	−1	2	0.90	0.50	98.59	88.12	84.56
4	−1	2	0.10	0.50	93.02	95.57	94.07
5	−1	2	0.50	0.90	98.66	96.67	97.24
6	0	2	0.50	0.50	97.15	88.31	91.67
7	−1	2	0.50	0.10	92.39	82.84	86.11
8	−1	2	0.50	0.90	98.66	96.67	97.24
9	0	2	0.50	0.50	97.23	88.36	91.69
10	−1	2	0.10	0.50	93.02	95.57	94.07
11	−1	2	0.50	0.10	92.39	82.84	86.11
12	−1	2	0.90	0.50	98.59	88.12	84.56
13	0	2	0.50	0.50	97.25	88.32	91.73
14	0	2	0.50	0.50	97.26	88.38	91.78
15	0	1	0.50	0.50	97.22	88.40	97.74
16	1	1	0.78	0.78	96.85	85.42	81.82
17	1	1	0.78	0.22	94.53	72.64	74.76
18	1	1	0.22	0.78	92.89	87.69	96.58
19	0	1	0.50	0.50	97.21	88.37	91.69
20	1	1	0.22	0.22	95.19	93.04	96.74
21	1	1	0.22	0.78	92.89	87.69	96.58
22	0	1	0.50	0.50	97.23	88.39	91.78
23	0	1	0.50	0.50	97.27	88.41	91.62
24	0	1	0.50	0.50	97.24	88.27	91.77
25	0	1	0.50	0.50	97.24	88.35	91.79
26	1	1	0.22	0.22	95.19	93.04	96.74
27	1	1	0.78	0.22	94.53	72.64	74.76
28	1	1	0.78	0.78	96.85	85.42	81.82

**Table 2:** Fatty acids profile of bigel and plain vegetable oil samples upon digestion via in vitro simulated gastrointestinal tract.

	Avocado Bigel		Avocado Oil		Coconut Bigel		Coconut Oil		Pomegranate Bigel		Pomegranate Oil	
Fatty Acid	Sample	Digested	Sample	Digested	Sample	Digested	Sample	Digested	Sample	Digested	Sample	Digested
C6	-	-	-	-	0.95 ± 0.08 ^a^	0.56 ± 0.00 ^b^	0.17 ± 0.00 ^a^	0.06 ± 0.02 ^b^	-	-	-	-
C8	-	-	-	-	16.39 ± 1.69 ^a^	10.71 ± 0.10 ^b^	2.26 ± 0.01 ^a^	2.43 ± 0.49 ^b^	-	-	-	-
C10	-	-	-	-	15.52 ± 1.77 ^a^	11.02 ± 0.33 ^b^	2.26 ± 0.01 ^a^	1.30 ± 0.06 ^b^	-	-	-	-
C12	-	-	-	-	161.12 ± 19.87 ^a^	119.45 ± 8.91 ^b^	23.62 ± 0.08 ^a^	0.92 ± 0.18 ^b^	-	-	-	-
C14	0.18 ± 0.12 ^a^	0.08 ± 0.02 ^a^	0.13 ± 0.00	n.d.	79.71 ± 10.05 ^a^	5.15 ± 0.00 ^b^	11.50 ± 0.04 ^a^	0.68 ± 0.09 ^b^	-	-	-	-
C16	41.28 ± 9.63 ^a^	23.96 ± 7.99 ^a^	64.50 ± 1.12 ^a^	9.30 ± 3.61 ^b^	93.49 ± 15.74 ^a^	59.63 ± 7.52 ^b^	5.33 ± 0.02 ^a^	0.36 ± 0.04 ^b^	19.87 ± 4.75 ^a^	10.76 ± 5.03 ^a^	6.44 ± 0.22 ^a^	1.16 ± 0.11 ^b^
C16:1 *c*7	0.21 ± 0.04 ^a^	0.16 ± 0.05 ^a^	0.32 ± 0.01 ^a^	0.11 ± 0.04 ^b^	-	-	-	-	-	-	-	-
C16:1 *c*9	8.94 ± 2.23 ^a^	5.22 ± 1.82 ^a^	14.95 ± 0.26 ^a^	1.75 ± 0.58 ^b^	-	-	-	-	-	-	-	-
C17	0.12 ± 0.02 ^a^	0.09 ± 0.03 ^a^	0.18 ± 0.00 ^a^	0.13 ± 0.06 ^a^	-	-	-	-	-	-	-	-
C17:1 *c*10	0.22 ± 0.05 ^a^	0.12 ± 0.04 ^a^	0.35 ± 0.00 ^a^	0.09 ± 0.01 ^b^	-	-	-	-	-	-	-	-
C18	9.49 ± 2.03 ^a^	6.69 ± 2.18 ^a^	7.89 ± 0.13 ^a^	2.64 ± 1.17 ^b^	86.26 ± 24.41 ^a^	0.08 ± 0.01 ^b^	2.34 ± 0.01 ^a^	0.17 ± 0.02 ^b^	19.65 ± 5.19 ^a^	9.80 ± 4.78 ^a^	5.18 ± 0.18 ^a^	0.77 ± 0.07 ^b^
C18.1 *t*12	-	-	-	-	-	-	-	-	0.28 ± 0.06 ^a^	0.20 ± 0.11 ^a^	0.12 ± 0.00 ^a^	0.11 ± 0.01 ^b^
C18:1 *c*9	132.01 ± 29.55 ^a^	70.27 ± 24.59 ^b^	227.40 ± 3.96 ^a^	27.13 ± 3.43 ^b^	53.29 ± 2.82 ^a^	0.53 ± 0.31 ^b^	3.42 ± 0.01 ^a^	0.24 ± 0.02 ^b^	36.62 ± 6.98 ^a^	18.39 ± 9.29 ^b^	12.60 ± 0.43 ^a^	0.10 ± 0.0 ^b^
C18:1 *c*11	9.89 ± 2.15 ^a^	5.26 ± 1.87 ^a^	16.77 ± 0.29 ^a^	1.87 ± 0.70 ^b^	-	-	-	-	1.65 ± 0.31 ^a^	0.67 ± 0.28 ^b^	0.93 ± 0.03 ^a^	n.d.
C18:1 *c*6	-	-	-	-	-	-	-	-	0.89 ± 0.16 ^a^	0.34 ± 0.15 ^b^	0.63 ± 0.02 ^a^	n.d.
C18:2 *t*9 *c*12	0.19 ± 0.04 ^a^	0.12 ± 0.04 ^a^	0.29 ± 0.00 ^a^	0.07 ± 0.00 ^b^	-	-	-	-	-	-	-	-
C18:2	26.47 ± 6.67 ^a^	15.95 ± 5.52 ^a^	44.16 ± 0.77 ^a^	7.03 ± 2.74 ^b^	3.93 ± 0.48 ^a^	2.46 ± 0.22 ^b^	0.54 ± 0.00 ^a^	0.06 ± 0.01 ^b^	19.83 ± 3.52 ^a^	8.06 ± 3.26 ^b^	12.37 ± 0.43 ^a^	0.06 ± 0.0 ^b^
*γ*C18:3	0.98 ± 0.25 ^a^	0.65 ± 0.19 ^a^	1.58 ± 0.03 ^a^	0.24 ± 0.08 ^b^	-	-	-	-	0.08 ± 0.03 ^a^	0.03 ± 0.00 ^a^	1.13 ± 0.04 ^a^	0.0.6 ± 0.01 ^b^
C20:1	0.79 ± 0.09 ^a^	0.38 ± 0.11 ^b^	1.39 ± 0.02 ^a^	0.13 ± 0.05 ^b^	-	-	-	-	-	-	-	-
C18:3	0.56 ± 0.05 ^a^	0.27 ± 0.09 ^b^	0.93 ± 0.02 ^a^	0.14 ± 0.07 ^b^	-	-	-	-	2.91 ± 0.58 ^a^	0.07 ± 0.03 ^b^	1.68 ± 0.06 ^a^	0.08 ± 0.01 ^b^
C22:1	-	-	-	-	-	-	-	-	0.50 ± 0.13	n.d.	0.04 ± 0.00 ^a^	n.d.
C18:3 *c*9 *t*11 *c*13	-	-	-	-	-	-	-	-	220.25 ± 37.40 ^a^	92.26 ± 41.31 ^b^	140.77 ± 4.86 ^a^	3.03 ± 0.78 ^b^
C18:3 *c*9 *t*11 *t*13	-	-	-	-	-	-	-	-	7.27 ± 1.01 ^a^	3.17 ± 1.29 ^b^	4.35 ± 0.15 ^a^	0.18 ± 0.15 ^b^
C18:3 *t*9 *t*11 *c*13	-	-	-	-	-	-	-	-	0.78 ± 0.26 ^a^	0.63 ± 0.32 ^a^	1.96 ± 0.07 ^a^	0.14 ± 0.12 ^b^
C18: 3 *t*9 *t*11 *t*13	-	-	-	-	-	-	-	-	3.61 ± 0.77 ^a^	0.64 ± 0.32 ^b^	0.49 ± 0.02 ^a^	0.09 ± 0.01 ^b^
C20:3	0.27 ± 0.03 ^a^	0.11 ± 0.03 ^b^	0.47 ± 0.00 ^a^	0.13 ± 0.01 ^a^	-	-	-	-	-	-	-	-
C20:5	0.18 ± 0.00 ^a^	0.12 ± 0.07 ^a^	0.29 ± 0.00	n.d.	-	-	-	-	-	-	-	-
C24	0.20 ± 0.03 ^a^	0.08 ± 0.03 ^a^	0.36 ± 0.00	n.d.	-	-	-	-	-	-	-	-
∑Fatty acids	231.99 ± 53.93 ^a^	129.51 ± 44.67 ^b^	381.96 ± 6.63 ^a^	50.77 ± 12.53 ^b^	510.67 ± 3.92 ^a^	209.59 ± 16.16 ^b^	51.45 ± 0.18 ^a^	6.19 ± 0.71 ^b^	334.48 ± 61.23 ^a^	145.10 ± 66.20 ^b^	188.68 ± 6.51 ^a^	6.95 ± 3.42 ^b^
∑SFAs	51.27 ± 11.76 ^a^	30.89 ± 10.23 ^a^	73.06 ± 1.27 ^a^	12.07 ± 4.84 ^b^	453.44 ± 6.70 ^a^	20.59 ± 16.88 ^b^	47.48 ± 0.17 ^a^	5.88 ± 0.73 ^b^	39.82 ± 10.02 ^a^	20.63 ± 9.84 ^a^	11.62 ± 0.40 ^a^	1.93 ± 0.18 ^b^
∑MUFAs	152.20 ± 34.17 ^a^	81.48 ± 28.52 ^b^	261.17 ± 4.54 ^a^	31.07 ± 4.81 ^b^	53.85 ± 2.58 ^a^	0.53 ± 0.31 ^b^	3.42 ± 0.01 ^a^	0.24 ± 0.03 ^b^	39.94 ± 7.64 ^a^	19.60 ± 9.83 ^a^	14.31 ± 0.45 ^a^	0.21 ± 0.01 ^b^
∑PUFAs	26.65 ± 6.97 ^a^	17.21 ± 5.94 ^a^	47.72 ± 0.82 ^a^	7.61 ± 2.88 ^b^	3.93 ± 0.21 ^a^	2.46 ± 0.27 ^a^	0.54 ± 0.01 ^a^	0.07 ± 0.01 ^b^	254.72 ± 4.57 ^a^	104.07 ± 46.52 ^b^	162.75 ± 5.61 ^a^	4.80 ± 3.61 ^b^

Results are expressed in mg/g and are the means of three determinations ± standard deviation. Values with different letters in the same line (for each oil) are significantly different, as determined by the one-way ANOVA test (*p* < 0.05). n.d. = not detected.

## Data Availability

The data presented in this study are available on request from the corresponding author.

## References

[1] McClements D.J., Öztürk B. (2021). Utilization of Nanotechnology to Improve the Handling, Storage and Biocompatibility of Bioactive Lipids in Food Applications. Foods.

[2] McClements D.J. (2015). Nanoscale Nutrient Delivery Systems for Food Applications: Improving Bioactive Dispersibility, Stability, and Bioavailability. J. Food Sci..

[3] Salminen H., Gömmel C., Leuenberger B.H., Weiss J. (2016). Influence of encapsulated functional lipids on crystal structure and chemical stability in solid lipid nanoparticles: Towards bioactive-based design of delivery systems. Food Chem..

[4] Shakeel A., Lupi F.R., Gabriele D., Baldino N., De Cindio B. (2018). Bigels: A unique class of materials for drug delivery applications. Soft Mater..

[5] Mao L., Lu Y., Cui M., Miao S., Gao Y. (2020). Design of gel structures in water and oil phases for improved delivery of bioactive food ingredients. Crit. Rev. Food Sci. Nutr..

[6] Martins A.J., Silva P., Maciel F., Pastrana L.M., Cunha R.L., Cerqueira M.A., Vicente A.A. (2019). Hybrid gels: Influence of oleogel/hydrogel ratio on rheological and textural properties. Food Res. Int..

[7] Shakeel A., Farooq U., Gabriele D., Marangoni A.G., Lupi F.R. (2021). Bigels and multi-component organogels: An overview from rheological perspective. Food Hydrocoll..

[8] Rehman K., Amin M.C.I.M., Zulfakar M.H. (2014). Development and Physical Characterization of Polymer-Fish Oil Bigel (Hydrogel/Oleogel) System as a Transdermal Drug Delivery Vehicle. J. Oleo Sci..

[9] Zheng H., Mao L., Cui M., Liu J., Gao Y. (2020). Development of food-grade bigels based on κ-carrageenan hydrogel and monoglyceride oleogels as carriers for β-carotene: Roles of oleogel fraction. Food Hydrocoll..

[10] Zhu Q., Gao J., Han L., Han K., Wei W., Wu T., Li J., Zhang M. (2021). Development and characterization of novel bigels based on monoglyceride-beeswax oleogel and high acyl gellan gum hydrogel for lycopene delivery. Food Chem..

[11] Zulfakar M.H., Chan L.M., Rehman K., Wai L.K., Heard C.M. (2017). Coenzyme Q10-Loaded Fish Oil-Based Bigel System: Probing the Delivery Across Porcine Skin and Possible Interaction with Fish Oil Fatty Acids. AAPS Pharm. Sci. Tech..

[12] Gumus C.E., Gharibzahedi S.M.T. (2021). Yogurts supplemented with lipid emulsions rich in omega-3 fatty acids: New insights into the fortification, microencapsulation, quality properties, and health-promoting effects. Trends Food Sci. Technol..

[13] Martinez R.M., Magalhães W.V., Sufi B.D.S., Padovani G., Nazato L.I.S., Velasco M.V.R., Lannes S.C.D.S., Baby A.R. (2021). Vitamin E-loaded bigels and emulsions: Physicochemical characterization and potential biological application. Colloids Surf. B Biointerfaces.

[14] Fayaz G., Goli S.A.H., Kadivar M., Valoppi F., Barba L., Calligaris S., Nicoli M.C. (2017). Potential application of pomegranate seed oil oleogels based on monoglycerides, beeswax and propolis wax as partial substitutes of palm oil in functional chocolate spread. LWT.

[15] Yu H., Shi K., Liu D., Huang Q. (2012). Development of a food-grade organogel with high bioaccessibility and loading of curcuminoids. Food Chem..

[16] Oh I.K., Lee S. (2018). Utilization of foam structured hydroxypropyl methylcellulose for oleogels and their application as a solid fat replacer in muffins. Food Hydrocoll..

[17] Hamdan M.A., Ramli N.A., Othman N.A., Amin K.N.M., Adam F. (2021). Characterization and property investigation of microcrystalline cellulose (MCC) and carboxymethyl cellulose (CMC) filler on the carrageenan-based biocomposite film. Mater. Today Proc..

[18] Amit, Jamwal R., Kumari S., Kelly S., Cannavan A., Singh D.K. (2020). Rapid detection of pure coconut oil adulteration with fried coconut oil using ATR-FTIR spectroscopy coupled with multivariate regression modelling. LWT.

[19] Amit, Jamwal R., Kumari S., Dhaulaniya A.S., Balan B., Singh D.K. (2020). Application of ATR-FTIR spectroscopy along with regression modelling for the detection of adulteration of virgin coconut oil with paraffin oil. LWT.

[20] Rehman K., Zulfakar M.H. (2017). Novel Fish Oil-based Bigel System for Controlled Drug Delivery and its Influence on Immunomodulatory Activity of Imiquimod Against Skin Cancer. Pharm. Res..

[21] Behera B., Singh V., Kulanthaivel S., Bhattacharya M., Paramanik K., Banerjee I., Pal K. (2015). Physical and mechanical properties of sunflower oil and synthetic polymers based bigels for the delivery of nitroimidazole antibiotic—A therapeutic approach for controlled drug delivery. Eur. Polym. J..

[22] Nasirpour-Tabrizi P., Azadmard-Damirchi S., Hesari J., Heshmati M.K., Savage G.P. (2020). Rheological and physicochemical properties of novel low-fat emulgels containing flaxseed oil as a rich source of ω-3 fatty acids. LWT.

[23] Rohman A., Irnawati, Erwanto Y., Lukitaningsih E., Rafi M., Fadzilah N.A., Windarsih A., Sulaiman A., Zakaria Z. (2021). Virgin Coconut Oil: Extraction, Physicochemical Properties, Biological Activities and Its Authentication Analysis. Food Rev. Int..

[24] Cervantes-Paz B., Yahia E.M. (2021). Avocado oil: Production and market demand, bioactive components, implications in health, and tendencies and potential uses. Compr. Rev. Food Sci. Food Saf..

[25] Drinić Z., Mudrić J., Zdunić G., Bigović D., Menković N., Šavikin K. (2020). Effect of pomegranate peel extract on the oxidative stability of pomegranate seed oil. Food Chem..

[26] Paul A., Radhakrishnan M. (2020). Pomegranate seed oil in food industry: Extraction, characterization, and applications. Trends Food Sci. Technol..

[27] Brodkorb A., Egger L., Alminger M., Alvito P., Assunção R., Ballance S., Bohn T., Bourlieu-Lacanal C., Boutrou R., Carrière F. (2019). INFOGEST static in vitro simulation of gastrointestinal food digestion. Nat. Protoc..

[28] Machado M., Costa E.M., Silva S., Rodriguez-Alcalá L.M., Gomes A.M., Pintado M. (2022). Pomegranate Oil’s Potential as an Anti-Obesity Ingredient. Molecules.

